# Implementing a Personalized Physical Therapy Approach (Coach2Move) Is Effective in Increasing Physical Activity and Improving Functional Mobility in Older Adults: A Cluster-Randomized, Stepped Wedge Trial

**DOI:** 10.1093/ptj/pzac138

**Published:** 2022-10-06

**Authors:** Ward Heij, Lieke Sweerts, J Bart Staal, Steven Teerenstra, Eddy Adang, Philip J van der Wees, Maria W G Nijhuis-van der Sanden, Thomas J Hoogeboom

**Affiliations:** Radboud University Medical Center, Radboud Institute for Health Sciences, IQ Health Care Nijmegen, the Netherlands; Radboud University Medical Center, Radboud Institute for Health Sciences, IQ Health Care Nijmegen, the Netherlands; Radboud University Medical Center, Radboud Institute for Health Sciences, IQ Health Care Nijmegen, the Netherlands; Musculoskeletal Rehabilitation Research Group, School for Allied Health, HAN University of Applied Sciences, Nijmegen, the Netherlands; Radboud University Medical Center, Radboud Institute for Health Sciences, Department of Health Evidence, Section Biostatistics, Nijmegen, the Netherlands; Radboud University Medical Center, Department of Health Evidence, Nijmegen, the Netherlands; Radboud University Medical Center, Radboud Institute for Health Sciences, IQ Health Care Nijmegen, the Netherlands; Radboud University Medical Center, Radboud Institute for Health Sciences, Department of Rehabilitation, Nijmegen, the Netherlands; Radboud University Medical Center, Radboud Institute for Health Sciences, IQ Health Care Nijmegen, the Netherlands; Radboud University Medical Center, Radboud Institute for Health Sciences, IQ Health Care Nijmegen, the Netherlands

**Keywords:** Aged, Decision Making: Clinical, Evidence-Based Practice, Patient-Centered Care, Physical Therapists, Primary Health Care

## Abstract

**Objective:**

The purpose of this study was to assess whether the superior cost-effectiveness of a personalized physical therapy approach (Coach2Move)—which was demonstrated in a previous trial compared with usual care physical therapy (UCP)—can be replicated in daily clinical practice.

**Methods:**

A multicenter, cluster-randomized, stepped wedge trial with 4 clusters consisting of 4 physical therapist practices in the Netherlands was used to compare a personalized physical therapy approach to elicit physical activity (Coach2Move) versus care as usual. Multilevel analyses for effectiveness were conducted for the amount of physical activity (Longitudinal Aging Study Amsterdam Physical Activity Questionnaire) and functional mobility (Timed “Up & Go” Test) at 3, 6 (primary outcome), and 12 months’ follow-up. Secondary outcomes were level of frailty (Evaluative Frailty Index for Physical Activity), perceived effect (Global Perceived Effect and Patient-Specific Complaints Questionnaires), quality of life (Euro Quality of Life-5 Dimensions-5 Levels [EQ-5D-5L]), and health care expenditures.

**Results:**

The 292 community-dwelling older adults with mobility problems visiting physical therapists were included in either the Coach2Move (n = 112; mean [SD] age = 82 [5] years; 60% female) or UCP (n = 180; mean [SD] age = 81 (6) years; 62% female) section of the trial. At baseline, Coach2Move participants were less physically active compared with UCP participants (mean difference = −198; 95% CI = −90 to −306 active minutes). At 6 months, between-group mean differences [95% CI] favored Coach2Move participants on physical activity levels (297 [83 to 512] active minutes), functional mobility (−14.2 [−21 to −8]) seconds), and frailty levels (−5 [−8 to −1] points). At 12 months, the physical activity levels of Coach2Move participants further increased, and frailty levels and secondary outcomes remained stable, whereas outcomes of UCP participants decreased. After the Coach2Move implementation strategy, physical therapists utilized significantly fewer treatment sessions compared with before the implementation (15 vs 22). Anticipated cost savings were not observed.

**Conclusion:**

This study replicated the results of an earlier trial and shows that Coach2Move leads to better mid- and long-term outcomes (physical activity, functional mobility, level of frailty) in fewer therapy sessions compared with UCP. Based on these and earlier findings, the implementation of Coach2Move in physical therapist practice is recommended.

**Impact:**

This article describes the implementation of the Coach2Move approach, a treatment strategy that has proven to be cost-effective in a previously conducted randomized controlled trial. Implementation of Coach2Move in a real-life setting allowed an evaluation of the effects in a clinically relevant population. Coach2Move has been shown to increase physical activity, improve functional mobility, and reduce frailty more effectively compared with UCP therapy and therefore has application for physical therapists working with older adults in daily clinical practice.

**Lay summary:**

Coach2Move is a new physical therapy approach for older adults. Implementation of Coach2Move in daily clinical practice can help people better outcomes over a longer period of time against similar costs compared with regular physical therapy.

## Introduction

Physical activity is beneficial to improve quality of life and to counter adverse effects of frailty and comorbidities in older adults.^1^ However, adoption and maintenance of an active lifestyle can pose a serious challenge, especially if an individual is dealing with health problems.^2^ Among older adults with mobility problems, poor health, fear of falling, and negative experiences of physical activity emerged as the most common barriers to being physically active.^3^ Physical therapists can play an essential role in problem analysis and promoting physical activity among older adults.^4^

**Figure 1:**
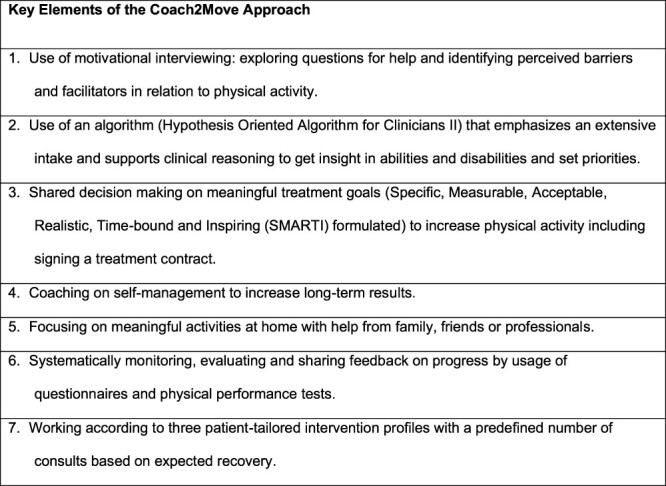
Key elements of the Coach2Move approach.

Coach2Move is a personalized physical therapy approach using an in-depth problem analysis resulting in personalized goals and tailored physical therapy treatment to improve physical activity.^5^ The key elements of Coach2Move are shown in Figure 1 and have been described in more detail in previous publications.^5–8^ A previously conducted randomized controlled trial (RCT) showed that Coach2Move is cost-effective compared with usual care physical therapy (UCP) for older adults with mobility problems.^8^ In the initial RCT, patients were randomized between therapists within selected practices, which led to drop-out of eligible participants as a result of their personal choice for a specific therapist.^7^ The primary researcher decided on in- and exclusion of patients and provided therapists feedback on the analysis and treatment process using electronic patient files.^8^ In agreement with recommendations from the UK Medical Research Council, we performed an implementation trial in daily clinical practice.^9^Table 1 shows a summary of the contrast between the initial effectiveness study (RCT) and the current implementation study (stepped wedge study).

**Table 1:** Contrast Between the Initial Coach2Move Effectiveness Study and the Current Coach2Move Implementation Study

**Elements**	**de Vries et al** ^8^ **(2016)**	**Heij et al (2022)**
Design	Randomized controlled trial with patients randomized over therapists	Stepped wedge, cluster-randomized trial with practices randomized over time
Practices	13	16
Therapists	Per practice: 1 general physical therapist (usual care), 1 specialized physical therapist in geriatrics*^a^* (trained in Coach2Move)	Per practice: At least 1 general physical therapist and 1 specialized physical therapist in geriatrics (both trained in Coach2Move)
Participants	130 community-dwelling older adults with mobility problems	292 community-dwelling older adults with mobility problems
Implementation	2 education days in motivational interviewing and Coach2Move Feedback per patient during treatment by research team Alternative electronic patient file alongside current patient file Reimbursement of prolonged intake by research team	E-assessment to determine baseline competencies 2 education days in motivational interviewing and Coach2Move Adaptations to the physical therapists’ own electronic patient files 3 Peer-assessment meetings Reimbursement of prolonged intake by health insurance
Delivery	1-on-1 coaching sessions with primary researcher	Peer group sessions led by primary researcher and education by peers in field
Outcomes	Primary: physical activity Secondary: frailty, walking speed and distance, mobility, health care costs, and quality of life at 3 and 6 mo	Primary: physical activity and functional mobility Secondary: frailty, perceived effect, health care costs, and quality of life at 3, 6, and 12 mo

^a^ Specialization refers to a therapist who holds a master’s degree in physical therapy in geriatrics.

The primary aim of the current trial was to assess whether Coach2Move is more effective than UCP in a pragmatic, real-world setting. A secondary aim was to evaluate costs and health-related quality of life of the Coach2Move implementation. Our hypothesis was that Coach2Move leads to better physical outcomes at reduced costs.

## Methods

### Study Design

**Figure 2:**
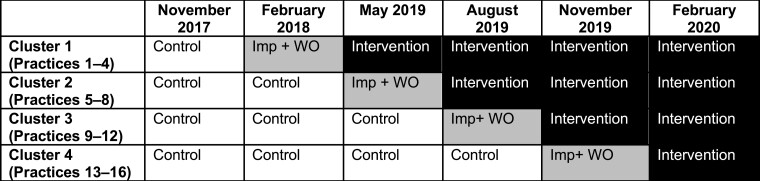
Depiction of stepped wedge, cluster-randomized design. Imp +WO = implementation and wash-out period.

We used a cluster-randomized, stepped wedge trial design in which we compared participants receiving UCP with participants receiving Coach2Move physical therapy. The trial has been prospectively registered on clinicaltrials.gov, and the protocol for this study was published in detail elsewhere.^10^ This study has been reported according to the CONSORT checklist.^11^ Within the stepped wedge design, Coach2Move was implemented in 4 randomized clusters (each containing 4 physical therapist practices) at standardized time points. This enabled comparison between and within clusters. The total study duration was 18 months (6 periods of 3 months) (see Fig. 2 for a visual depiction). Because 1 period was scheduled as a washout period (without including patients) between UCP and Coach2Move section, this resulted in 15 months of data collection. Each participant was measured at baseline (T0) and at 3 (T1), 6 (T2), and 12 (T3) months after inclusion in either the UCP or Coach2Move section. These participants’ measurements were assigned to the period and condition of their inclusion.

### Setting and Participants

Physical therapist practices in the Netherlands were eligible for participation if they (1) were primary care outpatient clinics; (2) had at least 1 general physical therapist with experience in working with older adults and 1 physical therapist specializing in geriatrics, as evidenced by a professional master’s degree; and (3) agreed to participate in education and peer-assessment activities. To ensure there were enough eligible patients for the study, practices had to have at least 75 newly registered older adults for physical therapy in the year prior to the study.

Individuals were eligible for inclusion if they were (1) ≥70 years of age and living independently, (2) experiencing limitations in purposeful physical movement of the body and/or had a physically inactive lifestyle (<30 min/d of moderate and/or vigorously physical activity) or were at risk of losing a physically active lifestyle in the near future, and (3) referred for physical therapy by a physician or decided to visit a physical therapist by themselves.

Exclusion criteria were if individuals were not ambulatory, in a palliative phase of an illness, likely to be institutionalized in the near future, or already receiving ongoing physical therapeutic treatment. Furthermore, individuals in an advanced stage of a severe degenerative neurological disease or those with a contraindication for being physically active or exercising were excluded from participation.

Written informed consent was obtained from all individuals or their informal caregivers.

Individuals with problems understanding advice and/or communicating with the physical therapist due to Dutch language problems and/or mild dementia were included.

### Study Procedures

Initially, all practices provided UCP. No instructions were provided on treatment content, frequency and/or duration of treatment episodes. To ensure there was no interference in the UCP treatment period due to the study, eligible individuals were visited by researchers within 1 week after registration for baseline measurements for conducting tests and completing questionnaires because they were not necessarily included in usual care. At some time point, based on cluster randomization, the implementation and washout period commenced. During this period, physical therapists were trained in applying the Coach2Move approach. Because this period was not used in the analyses, physical therapists were able to practice Coach2Move in daily practice.^10^ All baseline and follow-up measurements in both UCP and Coach2Move patients were conducted by 2 separate assessors (W.H. and L.S.), except for the baseline measurement during the Coach2Move phase (these were measured by the physical therapists as part of the Coach2Move strategy). Implementation of Coach2Move was based on a framework by Wensing and Grol,^12^ which consists of 5 phases: orientation, insight, acceptance, change, and sustainability. In the orientation phase, we piqued the interest of physical therapists and their practices by disseminating results from our previous trial through the publication of scientific articles and presentations at conferences. In the insight phase, we offered additional information about Coach2Move and the current study. In the acceptance phase, we visited the practices and discussed the impact implementing Coach2Move would have in their practice and what conditions need to be established. In the change phase, the practices transitioned from usual care to Coach2Move physical therapy. Lastly, in the sustain phase, we tried to facilitate the implementation of Coach2Move in daily clinical practice. A more thorough description of the framework was described in our protocol article.^10^

### Randomization, Masking, and Treatment Allocation

Coach2Move was rolled-out sequentially every 3 months to each cluster in a pre-assigned random order. The 4 practices for each cluster were selected based on similar geographical location (to ensure feasibility of measurements and educational sessions). Furthermore, practice characteristics (stand-alone physical therapist practice vs multidisciplinary health care centers) were distributed equally over the clusters by stratification. Randomization took place on cluster level by using a random number generator operated by an external independent researcher not involved in the study.

Because Coach2Move relies heavily on specific methods of history-taking and hypothesis-guided testing, resulting in a more time-consuming and extensive intake, it was therefore impossible to mask physical therapists. Furthermore, outcome assessors were also not masked due to the nature of the study. Nonetheless, each measurement was performed using valid and reliable standardized tests by the outcome assessors without information about earlier measurements or information from the therapist. Patients were masked for participating in the Coach2Move or UCP section during the study. Patients received an informative letter prior to participation in which the specific goal of the study (ie, comparing UCP vs Coach2Move) was not specified to make sure patient expectations were not biased by knowing group allocations.

### Implementation of Coach2Move

To implement Coach2Move, participating physical therapists attended 2 days of educational training (16 hours in total) and 3 peer-assessment meetings (9 hours in total).^10^ The first course day was dedicated to applying motivational interviewing. The second day involved education in other key elements of Coach2Move (Fig. 1). Before enrollment, physical therapists completed an electronic assessment (e-assessment, Medify, Amsterdam, the Netherlands) that measured their prior competencies with Coach2Move principles and their motivational interviewing skills. The outcomes of the e-assessment were used to tailor the 2-day predesigned education program to the needs and competencies of the participating physical therapists. Completion of the e-assessment was mandatory prior to participating in the Coach2Move course. In addition, electronic health records as used by the physical therapists were adjusted to the Coach2Move flow to support clinical reasoning, patient involvement, and process monitoring and to stimulate communication with local care networks if available. After the implementation period, physical therapists were expected to give treatment conforming to Coach2Move. The educational trajectory was free of financial costs. However, it required time and effort, for which the physical therapists received continuing education credits equivalent to 1 year of regular continuing vocational training by the Royal Dutch Association of Physical Therapy (KNGF). Physical therapists received financial reimbursement of additional time spent during the extensive intake, which was temporarily reimbursed by 6 of the nation’s largest health insurers.

### Outcomes for Functional Effectiveness and Costs

Primary outcomes for the effectiveness of Coach2Move on health outcomes were based on the physical activity level per week of patients as measured by the Longitudinal Aging Study Amsterdam Physical Activity Questionnaire (LAPAQ), subscale “moderate activity,” and the functional mobility as measured with the Timed “Up & Go” (TUG). For physical activity measurements, a change of 30 min/wk was considered as a clinically important difference.^13^ For the TUG, a change greater than the minimal detectable change of 2.08 seconds was considered as a meaningful change.^14^ Secondary outcomes included level of frailty (Evaluative Frailty Index for Physical Activity [EFIP]), perceived effect (Global Perceived Effect [GPE] and Patient-Specific Complaints [PSC] Questionnaires) and quality of life (EQ-5D-5L). For the EQ-5D-5L visual analog scale (VAS), a change of 8 points was considered clinically important.^15^ Outcomes were measured at 3-, 6-, and 12-month follow-up after inclusion in either the UCP or Coach2Move section.

Information on demographic characteristics, medical diagnosis, and living situation (alone or with partner) were extracted from physical therapy electronic health records together with information on adverse events and volume of care. At the patient level, costs were measured using costs questionnaires in which the patients and/or their family reported usage of health care utilities in the past 3 months.

### Sample Size and Data Analysis

The study’s primary endpoints were the outcomes on the TUG and the LAPAQ at 6 months. Effectiveness on health outcomes has been defined when at least 1 of the primary outcomes improves while the other does not deteriorate.^16^ The expected power of the combined objective for 360 patients was 86%.

Data analysis to determine overall effectiveness involved comparison of the data points of participants in the UCP section with participants in the Coach2Move section. In other words, patients and their measurements in a practice during a 3-month period represented the data points of that practice in that time period. The primary outcomes of those patients were determined as the change from baseline to 6 months. Because of the hierarchical design (patients nested within clusters, practices, and therapists), multilevel linear analysis was used to compare estimated means between UCP and Coach2Move participants with (nested) random effects for cluster, practice, and therapist.^16^ Given variances ${\sigma}_1^2,{\sigma}_2^2,{\sigma}_3^2$ at level 1 (older adults), at level 2 (physical therapist) and level 3 (practice), intracluster correlations were calculated as $\mathrm{IC}{\mathrm{C}}_{12}=\Big({\sigma}_2^2+{\sigma}_3^2\Big)/\Big({\sigma}_1^2+{\sigma}_2^2+{\sigma}_3^2\Big)$ and $\mathrm{IC}{\mathrm{C}}_{23}={\sigma}_3^2/\Big({\sigma}_2^2+{\sigma}_3^2\Big)$. These are the correlation of older adults within their physical therapist and the correlation of physical therapists within their practice.^17^ Time was included as a fixed effect.^18^ The analyses followed the intention-to-treat approach. Model fit was assessed from residual plots and observed versus model-predicted trajectories of clusters and practices over time.

The economic evaluation was performed from a health care perspective. For continuous (and assumed normal distributions) outcomes, we used a multilevel mixed-effects generalized linear model with random effect for cluster, practice, and therapist, which allowed repeated measures on individuals and fixed effect for each step while outcomes were adjusted for the systematically different observation periods and for clustering in the data.^19^ The EQ-5D-5L utility was corrected for baseline.^20^

All costs were calculated at each measurement point cumulating to a total number of costs over the full study duration. The cost analysis consisted of 2 parts. First, the direct number of UCP and Coach2Move sessions were determined on a per-patient basis. Moreover, volume of health care use and additional support and the number of health care–associated products were calculated per patient. These included visits to health care professionals and hospitals, use of medication, and use of domestic or nursing care.

The second part of the cost analysis consisted of determining cost prices for each volume of consumption for which the Dutch manual for costing in evaluation analysis was used.^21^ These were multiplied with the volumes of used health care. For resources where no guidelines or standard prices were available, cost prices were estimated. Quality-adjusted life years (QALY) were determined by applying the Dutch tariff to the EQ-5D-5L.^22^ The incremental cost-effectiveness ratio “cost per QALY gained” based on the EQ-5D-5L utility was computed, and uncertainty relating to clustering and design was determined. A cost consequence analysis was performed that assesses costs together with all secondary effect measures, including EQ-5D-5L utility scores of the Coach2Move intervention compared with UCP. All relevant health care costs between UCP and Coach2Move were compared during the evaluation period of 12 months.^22^

### Role of the Funding Source

The grant supporter of this study had no role in the design of this study nor in its execution, analyses, interpretation of the data, or decision to submit results.

## Results

**Figure 3:**
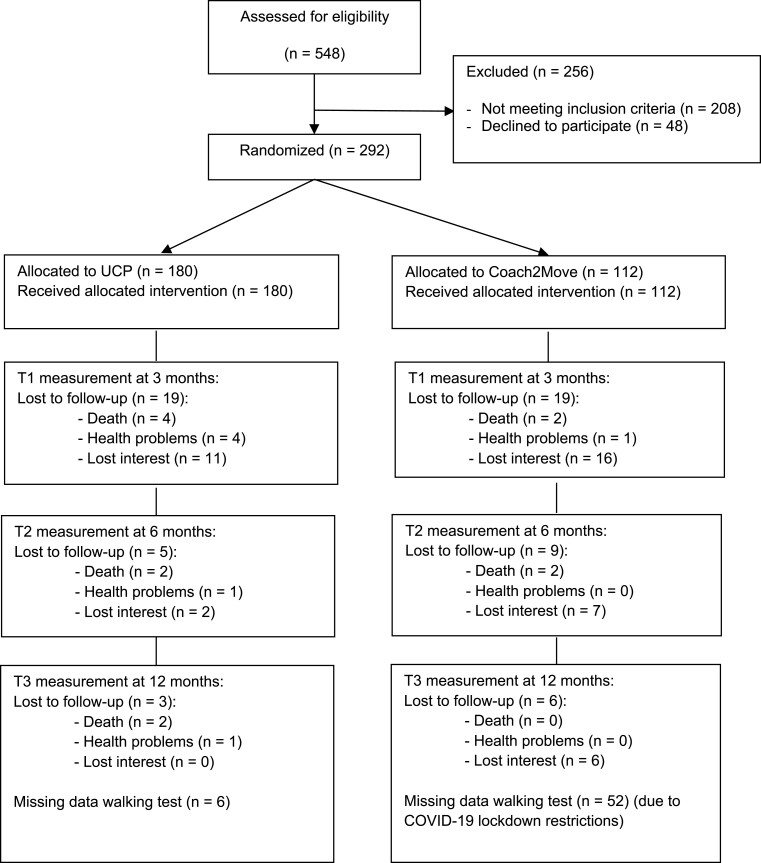
Overview of the patient flowchart.

Between September 2017 and May 2019, a total of 292 older adults participated in the study, of which 180 participants were included in the UCP section and 112 in the Coach2Move section of the study. The demographic characteristics of UCP and Coach2Move participants are described in Table 2. Statistical differences, as determined by the Mann–Whitney *U* test, were found in living situation and number of illnesses (*P* < .05); a higher percentage of participants receiving usual care lived independently at home during baseline and had more comorbidities. Follow-up responses in UCP and Coach2Move sections were 87% and 73% for the primary endpoint at 6 months, respectively (Fig. 3).

**Table 2:** Baseline Outcomes of Included UCP and Coach2Move Participants*^a^*

**Characteristic**	**Usual Care** **(n = 180)**	**Coach2Move (n = 112)**
Age, mean (SD), y	81.6 (6.6)	82.0 (5.8)
Female (as reported)	113 (62.8)	68 (60.7)
Marital status Single In a relationship Married Widow(er)	15 (8.3) 5 (2.8) 76 (42.2) 84 (46.7)	13 (11.6) 8 (7.1) 43 (38.4) 48 (42.9)
Education level Only primary education Practical education Vocational secondary education General secondary education University education	9 (5.0) 37 (20.6) 88 (48.9) 38 (21.1) 8 (4.4)	8 (7.1) 20 (17.9) 53 (47.3) 27 (24.1) 4 (3.6)
Living situation*^b^* Independently Sheltered housing Temporarily at nursing home	144 (80.0) 31 (17.2) 5 (2.8)	87 (77.7) 18 (16.1) 7 (6.3)
CIRS, mean (SD)*^b^*	7.8 (4.0)	6.2 (2.7)

^a^ Data are reported as number (percentage) of participants unless otherwise indicated. CIRS = Cumulative Illness Rating Scale; UCP = usual care physical therapy.

^b^ Statistically significant difference (*P* < .05).

Table 3 shows the mean scores on the LAPAQ, TUG, EFIP, EQ-5D-5L utility, PSC Questionnaire, and GPE Questionnaire of the Coach2Move participants compared with the UCP participants at each time point. At baseline, Coach2Move participants were more frail, had decreased functional mobility, were less physically active, and had a lower quality of life compared with UCP participants. Health outcomes (levels of frailty, functional mobility, and physical activity) of both UCP and Coach2Move participants improved in the first 3 months. Over the follow-up time periods, physical activity levels of Coach2Move participants increased at the different follow-up points, whereas physical activity levels among UCP participants decreased again. For functional mobility measured with the TUG, participants in the Coach2Move section improved over time. Quality of life of Coach2Move participants measured by the EQ-5D-5L VAS score improved over the follow-up time point. The perceived effect of Coach2Move participants as measured by the GPE and PSC increased over the follow-up time points.

**Table 3:** Mean Scores at Each Time Point and Effectiveness Outcomes of UCP Versus C2M Physical Therapy*^a^*

**Test**	**Type of Physical Therapy**	**T0 (Baseline)**			**T1 (3-Mo Follow-Up)**			**T2 (6-Mo Follow-Up)**			**T3 (12-Mo Follow-Up)**			**Mean Difference in Change Score From Baseline to T1 (95% CI)**	**Mean Difference in Change Score From Baseline to T2 (95% CI)**	**Mean Difference in Change Score From Baseline to T3 (95% CI)**
		**No. of Participants**	**Mean**	**SD**	**No. of Participants**	**Mean**	**SD**	**No. of Participants**	**Mean**	**SD**	**No. of Participants**	**Mean**	**SD**			
LAPAQ, min/wk	UCP	180	341.46*^b^*	574.36	161	349.08	419.48	156	337.61	437.98	153	233.12	303.60	Reference	Reference	Reference
	C2M	112	143.39*^b^*	364.67	93	270.96	308.13	82	366.83	412.04	76	422	494.62	136.92 (−89.67 to 363.21)	**297.44 (82.69 to 512.19)**	**441.56 (151.44 to 731.67)**
TUG, s	UCP	175	20.73	15.08	159	19.29	16.76	155	21.70	26.14	145	22.36	26.35	Reference	Reference	Reference
	C2M	109	22.54	19.23	90	17.0	9.18	79	15.90	8.84	24	20.57	14.04	**−7.82 (−12.88 to − 2.76)**	−**14.22 (−20.60 to − 7.84)**	**−17.39 (−29.15 to − 5.64)**
EFIP, range = 0–1	UCP	179	0.37	0.13	161	0.32	0.14	154	0.32	0.16	152	0.36	0.17	Reference	Reference	Reference
	C2M	107	0.39	0.13	92	0.33	0.14	81	0.30	0.16	76	0.28	0.14	**−3.36 (−5.61 to − 1.11)**	**−4.55 (−7.78 to − 1.31)**	**−8.86 (−12.42 to − 5.30)**
PSC, range = 0–10	UCP	179	6.22*^b^*	1.72	161	4.78	2.35	156	4.85	2.78	153	5.53	2.80	Reference	Reference	Reference
	C2M	107	7.74*^b^*	1.48	85	5.32	2.41	75	4.48	3.02	71	3.95	3.11	−**2.17 (−3.27 to − 1.07)**	−**2.45 (−4.05 to − 0.85)**	**−4.08 (−5.73 to − 2.43)**
EQ-5D-5L utility	UCP	179	0.63*^b^*	0.21	161	0.68	0.22	156	0.66	0.24	153	0.59	0.28	Reference	Reference	Reference
	C2M	110	0.53*^b^*	0.25	93	0.65	0.23	82	0.68	0.24	76	0.67	0.25	**0.16 (0.05 to 0.26)**	**0.18 (0.04 to 0.31)**	**0.29 (0.14 to 0.44)**
EQ-5D-5L VAS, range = 0–100	UCP	179	66.49*^b^*	14.87	161	69.15	11.93	156	67.76	12.03	153	63.39	15.40	Reference	Reference	Reference
	C2M	109	56.44*^b^*	14.92	93	68.27	13.53	82	72.83	12.27	76	71.21	12.29	**13.24 (6.66 to 19.81)**	**18.00 (10.46 to 25.54)**	**24.33 (15.81 to 32.85)**
GPE satisfaction subscale, range = 0–5	UCP				161	2.09	0.81	156	2.20	0.97	153	2.35	1.07		Reference	Reference
	C2M				92	1.88	0.98	82	2.05	0.97	76	2.12	0.86		0.28 (−0.37 to 0.93)	−0.01 (−0.77 to 0.74)
GPE effect subscale, range = 0–5	UCP				161	3.25	1.31	156	3.70	1.62	153	4.04	1.73		Reference	Reference
	C2M				92	2.79	1.18	82	3.09	1.53	76	3.47	1.45		0.11 (−0.25 to 0.46)	−0.10 (−0.53 to 0.33)

^a^ Values in bold type indicate a statistically significant difference (*P* < .05). C2M = Coach2Move; EFIP = Evaluative Frailty Index for Physical Activity; EQ-5D-5L = Euro Quality of Life-5 Dimensions-5 Levels; GPE = Global Perceived Effect; LAPAQ = Longitudinal Aging Study Amsterdam Physical Activity Questionnaire; PSC = Patient-Specific Complaints Questionnaire; TUG = Timed “Up & Go” Test; UCP = usual care physical therapy; VAS = visual analog scale.

^b^ Statistically significant difference at baseline (*P* < .05).

### Functional Effectiveness Analysis

For all longitudinal outcomes, the best model fit was considered the change from baseline. The ICCs for the change from baseline in the LAPAQ and the change from baseline in the TUG were consistent over the follow-up time points (3, 6, and 12 months). For the LAPAQ, the ICC for older adults within physical therapists was reasonably high (0.10–0.13), and the ICC for physical therapists within practices was high (0.5–1). For the TUG, the ICC for older adults within physical therapists was low (0–0.035), and the correlation of physical therapists within practices was high (1, when calculable) (see eAppendix 3, available at https://academic.oup.com/ptj). Missing data as a result of the COVID-19 restrictions was considered missing at random because of “missingness” being tied to external factors not associated with study outcomes. Therefore, missing (outcome) data were handled via the multilevel model under the missing-at-random assumption. Table 3 shows the results of the multilevel analyses corrected for baseline differences. Over the follow-up time points, there were statistically significant differences in favor of Coach2Move participants on minutes of physical activity per week at 6 months (+297 minutes; 95% CI = 83 to 512 minutes) and 12 months (+442 minutes; 95% CI = 151 to 732 minutes). In addition, Coach2Move participants had more statistically significant improvement in functional mobility at all follow-up points (mean differences at 3, 6, and 12 months = −8 seconds [95% CI = −13 to −3 seconds], −14 seconds [95% CI = −21 to −8 seconds], and − 17 seconds [95% CI = −29 to −6 seconds], respectively) than UCP participants. Coach2Move participants showed a larger decrease in frailty scores at all follow-up time points (mean differences at 3, 6, and 12 months = −5 points [95% CI = −6 to −1 points], −5 points [95% CI = −8 to −1 points], and − 9 points [95% CI = −12 to −5 points], respectively) than UCP participants. In addition, quality of life measured by the EQ-5D-5L VAS scores (mean differences at 3, 6, and 12 months = 13.24 points [95% CI = 6.66 to 19.81 points], 18 points [95% CI = 10.46 to 25.54 points], and 24.33 points [95% CI = 15.81 to 32.85 points], respectively) and PSC scores (mean differences at 3, 6, and 12 months = −2.2 points [95% CI = −3.3 to 1.1 points], −2.5 points [95% CI = −4.1 to −0.9 points], and −4.1 points [95% CI = −5.7 to 2.4 points], respectively) improved significantly more in Coach2Move participants over the follow-up points. The differences in outcomes on the GPE were not statistically significant between UCP and Coach2Move participants.

### Economic Evaluation

Coach2Move participants improved their EQ-5D-5L utility scores significantly more than UCP participants on all time points. Coach2Move participants received on average 15 physical therapy sessions versus 22 sessions in UCP (Suppl. Appendix 1). Over the full study duration, the difference from baseline on the EQ-5D-5L was 0.09 points (95% CI = −0.00 to 0.18 points) higher among the Coach2Move participants (Tab. 4). There were no statistically significant differences between health care costs of Coach2Move and UCP participants (mean difference: €800 [95% CI = −€1234 to €2834]) (see Suppl. Appendix 2). An incremental cost-effectiveness ratio for these outcomes suggests an extra cost of €8993 for a QALY gained.

**Table 4:** Cost-Utility Analysis of Coach2Move Versus Usual Care Physical Therapy

**Intervention**	**Estimated Marginal Mean Costs in Euros (95% CI)**	**Estimated Marginal Difference in Costs in Euros (95% CI)**	**Estimated Marginal Mean EQ-5D-5L Utility** **(95% CI)**	**Estimated Marginal Mean Difference in EQ-5D-5L Utility (95% CI)**
Usual care physical therapy	3374 (2239 to 4508)	Ref.	0.61 (0.55 to 0.68)	Ref.
Coach2Move	4174 (2506 to 5841)	800 (−1234 to 2834)	0.70 (0.62 to 0.79)	0.09 (−0.00 to 0.18)

## Discussion

This study evaluates the effectiveness on health outcomes and cost utilities of the personalized physical therapy approach Coach2Move compared with UCP in improving physical activity, functional mobility, and health outcomes of community-dwelling older adults. In the study, 282 community-dwelling older adults were included who had a mean age of 82 years old, were considered frail (EFIP score >0.2), had different levels of physical activity, and had considerable problems with functional mobility. Based on the results of the previous trial, it was expected that participants after intervention would become less frail and physically more active and would have less functional mobility problems regardless of the intervention they received (UCP and Coach2Move).^8^ The effects of the Coach2Move intervention on health outcomes, however, were expected to be sustained over a longer period of time. Moreover, it was expected that the effects of the Coach2Move intervention on health outcomes would be sustained over a longer period of time. The results confirmed that both interventions were effective in reducing frailty, increasing physical activity, and improving functional mobility at 3 months. However, our study shows that Coach2Move was more effective compared with UCP in decreasing frailty status and improving physical activity, functional mobility, patient-specific functional complaints, and quality of life not only in the short term but also the long term, whereas Coach2Move participants received fewer physical therapy sessions. Imbalances were observed for levels of frailty; however, after adjusting for frailty levels in a sensitivity analysis, effects were still consistent with the main analyses. Regarding cost-utility, an additional cost of €8993 per QALY for Coach2Move is considered acceptable in the Dutch context.^23^ We have tried to optimize the generalizability of the cost-utility results to health care systems of other countries by splitting the medical costs in frequencies per caregiver (Suppl. Appendix 1). This allows transferability to other systems.^24^ The anticipated cost reduction was not established because the costs were equal.

This study was a follow-up study as recommended by the UK Medical Research Council to assess if the effects on health outcomes and cost-effectiveness of the first trial could be replicated by implementing the approach in a pragmatic real-world setting.^8^^,^^9^ Both trials showed similarities in results: physical activity levels, functional mobility, patient-specific activities, and quality of life increased, while frailty decreased. An important finding is that in both studies, Coach2Move participants were able to increase their physical activity and health outcomes even further after completion of physical therapist treatment (3 months), whereas the physical activity levels and health outcomes decreased over time among UCP participants. In the previous trial, the follow-up period was 6 months, whereas in this study the results were similar with a prolonged follow-up of 12 months.^8^ Therefore, we conclude that Coach2Move is more effective in physical activity and functional mobility and thereby achieves better outcomes over longer follow-up durations compared with UCP.

A possible explanation for the nonsignificant, higher total costs of the Coach2Move intervention might be that at baseline, Coach2Move participants were more frequently living in a nursing home. In our study, older adults were eligible for inclusion if it was expected that they lived temporarily in a nursing home and would return home after rehabilitation. However, it appeared to be quite difficult for therapists to make a proper estimation if someone would stay temporarily or permanently (for instance, due to external factors like the COVID-19 pandemic). The number of participants who ended up staying permanently in a nursing home was higher among Coach2Move participants than among participants receiving UCP therapy (5% vs 1%, respectively). The latter likely resulted in disproportionally higher average costs compared with noninstitutionalized Coach2Move participants (€37,500 vs €9455, respectively). In sum, even though the direct physical therapy–related costs were less in the Coach2Move sample, the difference in housing likely explains the difference in overall costs.

### Comparison With the Literature

When comparing our findings with the previous Coach2Move trial, which included a less frail study population and a shorter follow-up duration (6 months), we found similar lasting effects on physical activity and frailty levels after the physical therapist treatment ended.^8^ Additionally, our current trial demonstrates that these positive outcomes on physical activity and frailty were maintained over an even longer follow-up (12 months). In line with the previous study, the cost-utility of the Coach2Move approach was still deemed acceptable, although the anticipated savings could not be realized.

There is a paucity of literature regarding the cost-effectiveness studies of physical therapist interventions in frail community-dwelling older adults.^25^ We identified a systematic review and 2 original research studies on this topic.^25–27^ The findings of a systematic review confirm that interventions that are tailored to the individual, like Coach2Move, are more likely to be cost-effective for frail older adults.^25^ Another review has concluded (added) physical therapy interventions are beneficial to health outcomes and prove to be a cost-effective alternative over usual care treatment (eg, surgery, medicinal treatment) in various conditions.^28^ These results are comparable with the outcomes from our study, because we performed an economic comparison between UCP treatment and Coach2Move. Two other original research studies demonstrated that physical exercise interventions supervised by physical therapists in frail community-dwelling older adults have a high probability for cost-effectiveness compared with usual care.^26^^,^^27^ In line with the Coach2Move approach, the studied interventions were multifactorial in nature and stimulated multidisciplinary care. Given the available literature and findings of this study, the evidence base is growing more and more compelling to restructure the physical therapy care for frail older adults. The Coach2Move approach might serve as a blueprint. A separate manuscript will describe the process analysis of the implementation of Coach2Move in daily clinical practice, including measurements of treatment adherence and adoption of the intervention by physical therapists.^10^

### Strengths and Limitations

A major strength of our study was that we applied broad inclusion and exclusion criteria based not on medical diagnoses but on problems in mobility and physical activities, which resulted in a representative study sample for daily clinical practice. Results from this study are therefore applicable in a real-life setting of physical therapist practice in older adults with mobility problems. By using a cluster-randomized stepped-wedge design, we have tried to decrease selection bias at the patient level to a minimum.^7^^,^^18^ In the previous trial, many older adults declined participation because they preferred their own therapist and did not want to be randomized to a therapist participating in the study.^8^ By randomizing clusters of practices, we have tried to overcome these objections by patients. However, a stepped wedge design has disadvantages over “classical” cluster RCTs.^29^ The nature of the design inherently increases the duration of the trial. In addition, due to the symmetric nature of the design, it was not possible to prolong the inclusion period of the design to make up for the lack of included participants in this trial due to external factors such as the COVID-19 pandemic.

Our study also has several limitations. First, the loss-to-follow-up rate was higher for Coach2Move participants (23%) compared with participants receiving usual care (15%). A possible explanation could be that patients in the Coach2Move section did not personally know the researchers when they were approached for an appointment for follow-up measurement at 3 months, whereas in the usual care section, the researchers contacted and visited participants at baseline. Often, participants were not receiving physical therapy treatment anymore, and in some cases there were misunderstandings about the purpose of the researcher’s visit after ending the physical therapy treatment. Second, the study did not reach the targeted sample size in the Coach2Move section. Much effort by the research team has been put into organizing a structural reimbursement scheme through the Dutch health care system for the prolonged Coach2Move intake. Alas, due to the abovementioned reasons, the finalization of this scheme was realized after several clusters implemented Coach2Move during the trial, resulting in uncertainty among participating physical therapists about whether including patients would be financially hazardous. This led to a lagging patient inclusion and (partly) caused an imbalanced distribution between UCP and Coach2Move participants.^30^ A process analysis discussing barriers and facilitators of the implementation of Coach2Move will be published later. Third, confounding by indication may have led to differences at baseline between Coach2Move and usual care participants, because older adults in the Coach2Move section were more frail, had less functional mobility, and perceived a lower quality of life. A more frail population is likely to receive more intensive physical therapist treatment.^31^

Fourth, although the used measurement tools were specifically designed for older adults and have good psychometric properties, the LAPAQ, which was used to measure time spent (in minutes) in physical activity, has been known to have a tendency to underestimate physical activity among older adults.^32^ As a result, the actual level of activity from either UCP or the Coach2Move intervention could be underestimated in daily clinical practice but do not bias differences in results between the groups. Fifth, outcome assessors were not masked for group allocation of participants because this would result in logistic complications for the planning of measurements due to the stepped wedge design. Sixth, and finally, during the last measurements of 52 participants in the Coach2Move section, we were faced with the COVID-19 pandemic. In the Netherlands this resulted in a complete lockdown and required older adults to isolate themselves. Older adults were encouraged to stay inside and reduce face-to-face interactions to a minimum. Therefore, we had to make amendments to our study protocol because we could not conduct measurements as usual and results could be biased. We left out the TUG performance test during these measurements because this would be impossible to conduct over the phone. Due to the consequences and uncertainty this global pandemic involved for our target population, a few participants chose not to continue their participation in the study. Some were physically and/or psychological affected by the consequences of the lockdown and felt stress and lost interest to continue their participation in the study.^33^ In addition, because older adults were asked to stay inside, their physical activity levels were affecting as well.^34^ Therefore, we asked participants about their physical activity levels before the outbreak of COVID-19 and how it differs from their situation now. Although we do not have evidence of a possible bias in our data, other studies have found this could possibly have resulted in recall bias, overestimating the treatment effect.^35^

Physical therapy according to the Coach2Move approach results in better outcomes (ie, minutes being physically active, functional mobility, frailty score, patient-specific complaints, and quality of life) against similar costs in frail community-dwelling older adults compared with UCP. Confounding by indication may have led to differences at baseline between Coach2Move and usual care participants, because older adults in the Coach2Move section were more frail, had less functional mobility, and perceived a lower quality of life. Regardless of the baseline differences, the cost-utility of the Coach2Move approach is considered acceptable and therefore we suggest implementation of Coach2Move into daily clinical practice.

## Supplementary Material

PTJ-2021-0844_R2_Suppl_Appendix_1_pzac138

PTJ-2021-0844_R2_Suppl_Appendix_2_pzac138

PTJ-2021-0844_R2_Suppl_Appendix_3_pzac138

## Data Availability

The datasets generated, analyzed, or both during the current study will be available from the corresponding author on reasonable request on the completion of the study.

## Clinical Trial Registration

This study is registered in the National Clinical Trials database (NCT03212859).
