# Supplementary material for: Implementing a Personalized Physical Therapy Approach (Coach2Move) Is Effective in Increasing Physical Activity and Improving Functional Mobility in Older Adults: A Cluster-Randomized, Stepped Wedge Trial
Source: Phys Ther. 2022 Oct 6;102(12):pzac138. doi: 10.1093/ptj/pzac138 (PMC10071485; doi:10.1093/ptj/pzac138)
Supplement: PTJ-2021-0844_R2_Suppl_Appendix_1_pzac138 [file ptj-2021-0844_r2_suppl_appendix_1_pzac138.pdf]

Supplementary Appendix 1. Frequencies of used resources.

Resource use	Usual care PT (n=180)		Coach2Move (n=112)		Unit cost (€)
	Patients that used resource number (%)	Units used mean (SD)	Patients that used resource number (%)	Units used mean (SD)	
Physical therapy		21.7		15.4	€30.69
<5 sessions	11 (7.1)	(15.3)	8 (9.8)	(10.3)	
5-12 sessions	61 (39.1)		41 (50.0)		
13-18 sessions	33 (21.2)		21 (25.6)		
>18 sessions	51 (32.7)		12 (14.6)		
General practitioner		5.2		4.4	€37.-
0 visits	17 (10.9)	(3.8)	9 (11.0)	(2.5)	
1 visit	29 (18.6)		13 (15.9)		
2 visits	20 (12.8)		13 (15.9)		
>2 visits	90 (57.7)		47 (57.3)		
Other allied healthcare practitioners	23 (18.4)	1.5 (4.8)	10 (18.5)	1.9 (4.5)	€34.-
Home care		81.9 (93.3)		41.5 (65.6)	
Housekeeping	125 (77.6)		71 (76.3)		€23.- per hour
Nurse	65 (40.4)		40 (43.0)		€56.60 per hour
Meal service	45 (28.0)	46.4 (85.8)	29 (31.2)	43.1 (87.1)	7.50 per meal
Outpatient care		4.1 (18.1)		5.5 (19.2)	
Nursing home admission	4 (2.5)		9 (9.7)		€43.40 per day

Temporary nursing home	1 (0.6)		1 (1.1)		€94.- per day
Temporary residential home	3 (1.9)		1 (1.1)		€150.- per day
Temporary geriatric rehabilitation	3 (1.9)		4 (4.3)		€515.- per day
Assistive devices		0.2 (0.5)		0.2 (0.5)	
Walker	17 (9.4)		17 (15.2)		€120.-
Crutches	4 (2.2)		1 (0.9)		€25.-
Cane	17 (9.4)		5 (4.5)		€23.-
Sole enhancement	1 (0.6)		1 (0.9)		€50.45
Other	2 (1.1)		3 (2.7)		€55.-
People who reported fall incidents:	78 (43.3)	1.17 (2.59)	27 (24.1)	0.45 (1.22)	
Hospital admissions:	29 (16.1)	0.19 (0.51)	9 (8.0)	0.10 (0.35)	
Deaths:	8 (4.4)		4 (3.6)		
