# Supplementary material for: Implementing a Personalized Physical Therapy Approach (Coach2Move) Is Effective in Increasing Physical Activity and Improving Functional Mobility in Older Adults: A Cluster-Randomized, Stepped Wedge Trial
Source: Phys Ther. 2022 Oct 6;102(12):pzac138. doi: 10.1093/ptj/pzac138 (PMC10071485; doi:10.1093/ptj/pzac138)
Supplement: PTJ-2021-0844_R2_Suppl_Appendix_2_pzac138 [file ptj-2021-0844_r2_suppl_appendix_2_pzac138.pdf]

Supplementary Appendix 2. Mean costs per patient at each timepoint.

		Usual care (n=180)	*Coach2Move (n=112)*
Costs medical specialist:	T1	160.8 (182.28)	146.59 (135.34)
	T2	123.06 (141.41)	113.84 (152.72)
	T3	133.02 (136.90)	103.79 (141.47)
Costs physical therapy:	T1	334.40 (182.08)	295.56 (176.37)
	T2 *	175.14 (225.40)	98.45 (161.02)
	T3 *	143.63 (187.52)	80.06 (161.42)
Costs other medical services:	T1	49.17 (110.59)	56.83 (87.67)
	T2	43.43 (96.89)	45.13 (94.84)
	T3	53.91 (119.02)	44.71 (70.54)
Medication costs:	T1	236.83 (330.50)	352.88 (1142.27)
	T2	238.84 (331.18)	396.34 (1218.08)
	T3	228.91 (264.34)	411.17 (1261.89)
Costs of operations:	T1 *	276.12 (1135.85)	54.78 (528.33)
	T2	114.36 (674.90)	118.17 (754.04)
	T3	170.16 (933.11)	110.66 (836.61)
Costs hospital admissions:	T1	301.86 (1647.71)	399.13 (1816.51)
	T2	595.38 (3190.37)	171.22 (921.26)
	T3	370.07 (1527.91)	563.48 (2144.82)
Costs emergency ward:	T1	13.70 (73.43)	23.97 (83.98)
	T2	18.17 (73.68)	23.05 (82.54)
	T3	17.62 (72.65)	34.24 (99.13)
Costs domestic help:	T1	416.43 (340.00)	476.25 (314.47)
	T2	452.04 (338.60)	420.45 (299.95)
	T3 *	489.75 (331.21)	327.47 (277.57)
Costs nursing care:	T1	612.02 (1178.03)	737.34 (1171.97)
	T2	452.04 (338.60)	637.10 (1251.37)

	T3	489.75 (331.21)	762.44 (1228.38)
Costs daycare:	T1	85.71 (303.45)	70.68 (263.77)
	T2	123.25 (360.81)	53.51 (242.07)
	T3	152.29 (401.26)	79.25 (537.51)
Costs meal service:	T1	114.46 (226.49)	163.21 (255.01)
	T2	132.64 (240.15)	163.54 (261.20)
	T3	173.18 (262.08)	251.60 (293.03)
Costs nursing home:	T1 *	204.97 (1518.05)	37.50 (359.69)
	T2	0	0
	T3 *	19.93 (203.94)	97.83 (663.49)
Costs rehabilitation center:	T1 *	0	576.68 (4147.39)
	T2	505.10 (4248.20)	527.56 (4777.27)
	T3	277.31 (2090.39)	548.59 (3720.70)
Costs residential care:	T1 *	159.98 (1064.31)	522.11 (1866.85)
	T2 *	166.97 (1075.59)	914.60 (2550.55)
	T3 *	266.22 (1439.00)	1195.44 (2911.99)
Costs modification to housing:	T1	92.98 (398.98)	113.55 (388.67)
	T2	23.59 (170.23)	29.02 (222.98)
	T3	119.74 (522.32)	51.05 (287.43)
Costs medical devices:	T1	182.23 (478.73)	100.27 (390.75)
	T2 *	175.06 (457.14)	48.23 (189.11)
	T3	77.00 (319.51)	35.39 (142.20)
Costs walking aids:	T1 *	2.68 (19.49)	12.22 (75.48)
	T2 *	0	2.16 (2.16)
	T3	0	0
Total costs:	T1	3247.76 (4209.57)	4103.30 (6258.43)
	T2	3712.12 (7619.88)	3668.20 (6978.19)
	T3	3458.58 (4602.70)	3233.18 (6279.43)

***An asterisk indicates a statistical significant difference (p < 0.05).**
