# Supplementary material for: Implementing a Personalized Physical Therapy Approach (Coach2Move) Is Effective in Increasing Physical Activity and Improving Functional Mobility in Older Adults: A Cluster-Randomized, Stepped Wedge Trial
Source: Phys Ther. 2022 Oct 6;102(12):pzac138. doi: 10.1093/ptj/pzac138 (PMC10071485; doi:10.1093/ptj/pzac138)
Supplement: PTJ-2021-0844_R2_Suppl_Appendix_3_pzac138 [file ptj-2021-0844_r2_suppl_appendix_3_pzac138.pdf]

Supplementary Appendix 3. ICC Calculations

	Variance elderly	Variance physical therapists	Variance practices	Correlation of elderly within physical therapist	Correlation of physical therapists within practice
LAPAQ 6 months	162964	2551	15842	0.10	0.86
TUG 6 months	186	0	0	0	Not calculable
LAPAQ 3 months	203014	0	22871	0.10	1
TUG 3 months	115.7	0	4.2	0.035	1
LAPAQ 12 months	276397	21277	21735	0.13	0.5
TUG 12 months	405.6	0	11.3	0.027	1
LAPAQ = Longitudinal Aging Study Amsterdam Physical Activity Questionnaire; TUG = Timed Up and Go;					
